# A twin disaster: Addressing the COVID-19 pandemic and a cerebrospinal meningitis outbreak simultaneously in a low-resource country

**DOI:** 10.1080/16549716.2020.1795963

**Published:** 2020-08-07

**Authors:** Samuel Adjorlolo, Daniel Lawer Egbenya

**Affiliations:** a Department of Mental Health Nursing, School of Nursing and Midwifery, University of Ghana, Legon, Ghana; b Research and Grant Institute of Ghana, Accra, Ghana; c Department of Anatomy and Cell Biology, School of Medical Sciences, College of Health and Allied Sciences, University of Cape Coast, Cape Coast, Ghana

**Keywords:** COVID-19, cerebrospinal meningitis, healthcare resources, equity, LMIC

## Abstract

Managing a deadly pandemic in low- and middle-income countries (LMIC) is challenging. The task becomes tougher when there is an outbreak of an equally deadly disease. This is the present situation of Ghana, a low-resource country, that is confronted with the coronavirus disease 2019 (COVID-19) pandemic and cerebrospinal meningitis (CSM) outbreak. Apart from the resource constraint at both governmental and individual levels, such a situation affects the overall wellbeing of ordinary citizens as well as healthcare professionals, particularly those in high-risk areas. Perhaps, more than ever, we have to ensure equitable distribution of scarce healthcare resources in our effort to manage this ‘twin disaster’ of COVID-19 and CSM. We evaluated Ghana’s situation (outbreak response) and recommended measures to help us navigate this conundrum of a public health crisis.

## Background

Confronting two deadly infectious diseases in low- and middle-income countries (LMIC) has severe implications for the quality of healthcare provided and overall wellbeing of the people. This is hugely due to the inadequate healthcare personnel and poor healthcare infrastructure in these countries. This is the case of Ghana that is presently battling with the coronavirus disease 2019 (COVID-19) and cerebrospinal meningitis (CSM), two deadly infectious diseases. We examined how the response strategies being adopted in Ghana may affect management outcome of both diseases and what can be done differently, using scarce national and local resources, to improve the management of these infectious diseases.

Within a spate of about three months, COVID-19 which started as an outbreak in Wuhan, China in late December 2019, became a pandemic. It is caused by the novel coronavirus called Severe Acute Respiratory Syndrome Coronavirus (SARS-CoV-2) [1]. As at 12 May 2020, 00:00 GMT, globally, there are 4,088,848 COVID-19 confirmed cases and 283, 153 deaths in 215 countries and territories [2] with 1,471,496 recoveries [3]. Ghana has recorded 5, 127 confirmed COVID-19 cases, 494 recoveries and 22 deaths as at 12 May 2020 [4]. These cases are confined to the two biggest cities (and their surrounding towns) of Ghana: Accra and Kumasi. This corresponds to a national case fatality rate of ~0.4%, lower than the global average of ~7%. Most of those who died were said to be old and had underlying comorbidities including diabetes and hypertension. In April 2020, 60% of COVID-19-infected persons were males. Following the confirmation of COVID-19 cases in the country, the government of Ghana and relevant State institutions; namely, Ghana Health Service and Ministry of Health, have activated the country’s public health response system to manage the pandemic. At the same time, there was an outbreak of CSM in the northern part of the country.

CSM is an infectious disease that is marked by inflammation of the meninges (membranous coverings of the brain and the spinal cord). Agents such as bacteria, virus, fungi and parasites cause CSM. Bacterial CSM, the commonest type of CSM, is caused mainly by *Neisseria meningitidis, Streptococcus pneumoniae*, and *Haemophilus influenza* [5–7]. Ghana falls within the African CSM belt that stretches from Senegal to Ethiopia. The CSM annual outbreak in Ghana occurs from November to May/June during the dry weather periods of northern Ghana (Northern, North East, Savanna, Upper East and Upper West Regions); a season characterised by cold wind and enormous dust [8–10]. Sporadic cases are also reported in parts of the then Brong Ahafo and upper part of the Volta regions.

Acute bacterial meningitis is among the leading cause of disability in LMICs and has a higher prevalence rate in LMICs than in high-income countries [7,11]. A five-year evaluation study on CSM in Ghana, spanning the period 2010–2015 [12], reported that 1,176 cases have been recorded in the northern part of the country. Similarly, that from December 2015 to February 2016, 133 laboratory-confirmed cases of meningitis (46.5% of suspected cases within the period) were reported in the then Northern, Upper East and Upper West regions of the country [6]. From January 2008 to December 2010, 163 confirmed cases of meningitis were found [11]. In 2016, there were about 90 deaths and major morbidities that resulted from about 400 suspected cases of meningitis in Ghana [9]. With an overall mean age of 23.7 years, about 53% of suspected cases of CSM were males and the remaining 47% were females [9] implying an almost proportionate infection rate by gender [12]. Additionally, the researchers reported that from October 2016 to January 2017, 285 of a 330 CSF sample from 41 districts in the northern zone of Ghana tested positive for bacterial meningitis, depicting a positivity rate of 86%. Mortality rate of CSM in Ghana is between 36% and 50% [13,14].

This year, more than 400 confirmed CSM cases with about 50 deaths in the country’s CSM endemic regions: Northern, North East, Savanna, Upper East and Upper West Regions, have been recorded [15,16]. The recent outbreak, particularly in the Upper West Region, has the highest number of CSM fatalities and it is caused by a novel strain, *Neisseria meningitidis* serotype X, and *Streptococcus pneumoniae* which has a mean case fatality rate of 40% [15]. Just like SARS-CoV-2, this novel strain (*Neisseria meningitidis* serotype X) has no vaccine. The Upper West region has about 15% CSM case fatality rate resulting from 258 CSM cases and 40 deaths. Overall, at the moment, the national data suggests a higher case fatality rate of CSM than COVID-19. This notwithstanding, not much governmental effort, for instance in terms of personnel and material resource allocation, has been channelled to combating the more deadly CSM outbreak. This is contrary to past experiences in CSM management in which control measures, case management, contact tracing and public education on identification of early signs and symptoms were vigorously and timeously carried out [6,8].

COVID-19 and CSM have similar signs and symptoms including fever, headache and confusion or altered consciousness [6,8]. Additionally, CSM is characterised by neck stiffness, vomiting and photophobia [8,17]. Both diseases have been shown to result in some level of hearing loss, cognitive challenges and brain damage [12,18,19]. Specifically, neurological challenges including epilepsy and mental retardation have been reported in survivors of CSM [5,7]. Both diseases may spread through air droplets, saliva and respiratory secretions when people are in close contacts [12,20]. Their incubation periods are also similar and is estimated to be about 2–10 days [20,21].

## The prevailing ‘twin disaster’ in Ghana: COVID-19 and CSM

The management of COVID-19 and CSM in Ghana portrays a case of inequity in healthcare resource distribution. Equity in healthcare resource distribution requires that such resources are shared to people based on the extent of their needs of those resources [22,23]. Since the confirmation of COVID-19 in Ghana, a high-level governmental involvement in confronting the disease has been shown. This has culminated in a number of interventions aimed at preventing, curbing the spread and treatment of the disease. These include the closure of the country’s entry ports, enactment of imposition of restrictions law which resulted in lockdowns in parts of the country, demarcation of isolation and treatment centres, setting up of a national COVID-19 trust fund, acquisition of a one billion dollar bailout from the International Monetary Fund (IMF) and initiation of enhanced contact tracing and testing. This is partly due to the severe havoc being caused by COVID-19 in other countries particularly in Europe and the United States. Unfortunately, in Ghana, these efforts targeted at COVID-19 have resulted in CSM being pushed to the background while it causes much devastation in the outbreak areas. The activation of meningitis surveillance by the Ghana Health Service and deployment of its Rapid Response Team appeared late and ineffective [24,25]. Additionally, Regional and District Public Health Emergency Management Committees were activated and provision of some medical supplies was carried out. However, these Teams had little or no resources to work with as healthcare officials and local authorities continued to appeal to the government and philanthropists for resources to combat the outbreak [26]. This is in stark contrast to the equity requirement in healthcare resource distribution. Equity in the distribution of healthcare resources ensures that people have access to the needed healthcare services hence improved management of medical conditions [27,28]. Inequality exists in Ghana’s healthcare system and has been largely driven, over the years, by urban-rural geographical differences [29,30]. The differences exist in the distribution of healthcare services and resources (facilities and personnel). However, the markedly differences in response strategies in the two present outbreaks might be due to the severe danger being caused by COVID-19 in other countries as well as the probability of spread of COVID-19 to all other parts of the country compared to CSM whose likelihood of spread throughout the country is lower.

While available evidence suggests that the aged are at higher risk of both infection and death resulting from COVID-19, children and young adults suffer much more from CSM [5,8,11,12,31–33]. This suggests that the entire population, particularly in the CSM endemic areas, maybe at a higher risk of infection of this ‘twin disaster’ – COVID-19 and CSM. Also, the CSM endemic areas are largely rural settings characterised by inadequate healthcare personnel and supplies such as drugs hence people are likely to receive poor medical care. Additionally, having to live through these diseases might come with psychological distress as people might be living their lives in fear and anxiety not knowing if they will survive these infectious diseases.

A challenge associated with dealing with CSM is its diagnosis especially in the areas experiencing the outbreak [9]. In the management of CSM, early diagnosis contributes to better treatment outcome [9,15]. Most of the few health facilities are health centres/posts and Community-based Health Planning and Services (CHPS) compounds which are meant to provide basic medical care [34,35]. Since these facilities lack the requisite equipment and professionals, misdiagnosis of CSM becomes a likely occurrence [36,37]. To complicate matters, because CSM and malaria have similar signs and symptoms, there may be misdiagnosis of CSM as malaria, a very common tropical disease in Ghana. This implies that persons suffering from CSM may be triaged as malaria patients, increasing the likelihood that they may be out there in the public infecting others. This is particularly the case for persons with CSM but who are asymptomatic.

The lack of adequate personal protective equipment (PPE) for healthcare workers in the CSM endemic zones may create an increased sense of psychological distress among these workers. This becomes worse because on a daily basis, healthcare professionals throughout the country are calling for more PPE to fight COVID-19. Each day, as healthcare officials in the CSM endemic zones report for work, they are confronted with the thought of working in an environment where they are prone to be infected with two highly contagious diseases. Should they be infected with any of these two diseases, their immediate family members who they share rooms with at home are at high risk of being infected too.

The ‘twin disaster’ of COVID-19 and CSM are having a huge toll on the livelihood of people in the CSM endemic areas. The area is, largely, a rural setting that forms part of the high poverty-ridden areas of the country. The many COVID-19 restrictions instituted by the government such as social distancing, some of which are also beneficial to preventing the spread of CSM, are affecting the livelihoods or work activities and finances of most of these people. This is likely to affect their overall preparedness to adhere to measures aimed at fighting off both the pandemic and the outbreak.

## The way forward

Despite the acknowledgement of COVID-19 as a pandemic with its attendant consequences, the available data, presently, showed that CSM is equally catastrophic, especially in Ghana. Immediate strong governmental effort should be channelled towards the fight against CSM in the outbreak areas in the country. This will ensure that equitable distribution of healthcare resources will be adhered to. The government, through the Ghana Health Service, should increase its interventions in the area, including stockpiling of drugs such as antibiotics, intravenous fluids and laboratory CSM test kits (reagents, etc.). Having commenced local production of PPE aimed at fighting COVID-19, an increased effort should be made in this regard to make a lot more available for health professionals in the CSM outbreak areas. Indeed, available evidence suggests that many healthcare professionals are getting infected with COVID-19, both in Ghana and the world at large [38–41]. To boost the confidence of healthcare professionals combating this ‘twin disaster’ as well as to ensure that they are not unnecessarily put at risk, it is recommended that enough PPE are made available to them. It is important to state that since both infectious diseases have a number of similarities, resources aimed at managing COVID-19 could be leveraged upon to fight CSM as well. When equity in healthcare resource distribution is ensured, there is better disease management and overall outcome [42].

Having known the exact strain causing the current outbreak, case definitions of CSM diagnosis should be updated. Consequently, healthcare officials, at all levels, in these areas should be informed of the needed update.

Having conducted about 150 thousand tests for COVID-19 presently, available data showed that by 8 May, Ghana was among the leading countries in case testing per capita in Africa [43,44]. This is partly attributed to the effective contact tracing being employed. It is also recommended that the country should, as a matter of urgency, deploy same approach to trace persons who have come into contact with CSM positive persons and test these people for CSM. This becomes imperative owing to possible asymptomatic CSM cases.

Similarly, apart from the need for the central government to provide enough funding, the nation’s health insurance authority must ensure prompt processing and payment of claims submitted by healthcare facilities managing CSM cases. This will enable the facilities to effectively acquire other needed essentials to ensure effective and efficient running of their activities. Additionally, just as for COVID-19 treatment, CSM treatment should be entirely free of charge, at all times. This will encourage the already economically challenged persons in the CSM endemic areas to avail themselves for medical treatment should they have signs and symptoms of CSM.

Apart from dealing with the healthcare facilities and personnel-related issues, public awareness creation or education on CSM is critical. This is because lack of knowledge on early signs and symptoms of a disease affects treatment outcome [10]. Early case detection leads to early diagnosis and treatment [9]. Therefore, intense community-level public education on the prevention and curbing the spread of CSM should be carried out in the endemic areas. The need for early report of signs and symptoms of CSM to healthcare facilities, avoiding overcrowding and practising good personal hygiene should be emphasised in the campaign. This should be done not only by healthcare professionals but also by traditional and opinion leaders and NGOs operating in the outbreak areas. The use of local authorities in the awareness creation will enhance community buy-ins and adherence to the preventive measures. It can be done through the use of vans fitted with public address systems in which live and pre-recorded educational messages on CSM are broadcast. Equally, most local communities in Ghana now have public address platforms fixed at a central location in the community which can be used. Public education will also reduce misinformation about CSM; some of which could account for the refusal of people to seek early medical care. Importantly, this community-level education should be an annual programme that should be carried out prior to the start of the CSM outbreak period in the five northern-most regions of Ghana as well as the middle zones.

For the medium- to long-term, the traditional housing facility in the most endemic areas which pose as favourable grounds for the spread of CSM due to poor ventilation should be improved. These houses, apart from their poor ventilation, are highly populated with many inhabitants owing to the communal living of Ghanaians as well as the high level of poverty in most of these areas. Government and other stakeholders should institute a modern and affordable housing scheme for the indigenes to tackle this.

People living in the CSM endemic regions of Ghana are likely to be experiencing some level of psychological distress having to be fighting both COVID-19 and CSM simultaneously. This is because unlike CSM which is endemic in particular areas of the country, COVID-19, with the passage of time, is more likely to spread to other parts of the country. The psychological distress might be in the form of anxiety, depression, insomnia and the likes. This might affect their mental health and overall wellbeing. In this light, it is recommended that health authorities should engage the services of the Ghana Psychological Association and other professional bodies to help reduce the psychological distress people in the CSM areas might be going through in these times.

## Conclusion

A developing country confronted with two deadly diseases at the same time requires planned and concerted efforts to use its meagre resources, both human and material, to successfully tackle the diseases. We are presently doing quite well in managing the COVID-19 pandemic while doing little to combat the more deadly CSM, per our local case fatality rate. There is enormous similarity between both diseases and so it is recommended that we apply measures and effort that can help us achieve success at preserving lives and reducing the harmful effects of the two diseases on Ghanaians. Much effort should be paid to ensure equitable distribution of healthcare resources with an overarching aim of effectively managing the disease and improving overall outcome.

## Paper context

The coronavirus disease pandemic has severely stretched the healthcare system of many countries. Simultaneously confronting an outbreak of an even deadlier infectious disease, cerebrospinal meningitis, in a low-resource country with already compromised healthcare system and infrastructure, is a major challenge. Effective management of these conditions to ensure better outcome and reduced psychological distress among the population require equitable distribution of scarce healthcare resources and public education suited to the peculiarities of such a developing country.
